# Case report: Flow changes in routes of collateral circulation in patients with LVO and low NIHSS: a point favor to treat

**DOI:** 10.3389/fneur.2023.1165484

**Published:** 2023-06-08

**Authors:** Elizeu Pereira dos Santos Neto, Ícaro Araújo de Sousa, Arthur de Oliveira Veras, Marx Lima de Barros-Araújo, Irapuá Ferreira Ricarte, Octávio Marques Pontes-Neto

**Affiliations:** ^1^Institute of Radiology, University of São Paulo School of Medicine, Hospital das Clínicas, São Paulo, SP, Brazil; ^2^Neurologist and Interventional Neuroradiologist, Hospital Santa Maria, Teresina, PI, Brazil; ^3^Department of Neuroscience and Behavior Sciences, Medical School of Ribeirão Preto, University of São Paulo, Ribeirão Preto, SP, Brazil; ^4^Department of Neurology and Neurosurgery, São Paulo Federal University, São Paulo, SP, Brazil

**Keywords:** low NIHSS score, collateral circulation, large vessel occlusion (LVO), acute ischemic stroke (AIS), transcranial Doppler ultrasound, endovascular thrombectomy

## Abstract

The effectiveness of endovascular thrombectomy in patients presenting low National Institutes of Health Stroke Scale (NIHSS) scores remains controversial, and the acquisition of additional evidence is required to refine the selection of candidates who may benefit the most from this therapeutic modality. In this study, we present the case of a 62-year-old individual, with left internal carotid occlusion stroke and low NIHSS, who had compensatory collateral flow from Willis polygon via the anterior communicating artery. The patient subsequently exhibited neurological deterioration and collateral flow failure from Willis polygon, indicating the need for urgent intervention. The study of collaterals in patients with large vessel occlusion stroke has garnered considerable attention, with research suggesting that individuals with low NIHSS scores and poor collateral profiles may be at a heightened risk of early neurological deterioration. We postulate that such patients may derive significant benefits from endovascular thrombectomy, and may posit that an intensive transcranial Doppler monitoring protocol could facilitate the identification of suitable candidates for such intervention.

## 1. Introduction

Acute ischemic stroke (AIS) caused by large vessel occlusions (LVOs) requires emergency detection in prehospital screening, and a rapid diagnosis along with treatment is essential to reduce morbidity and mortality (1). Clinical rating scales have been published to assist in the identification of LVO stroke and guide clinical decisions, but no single scale has demonstrated clear superiority, and few are used in practice consistently (2).

The diagnosis is made by clinical and radiological approaches/methods, with endovascular thrombectomy (ET) becoming the standard of care as a result of randomized trials that showed improved functional outcomes in patients who had thrombectomy with successful recanalization. However, several important factors represent crucial information for treatment decision-making, such as the size of the core infarct, the volume of recoverable penumbra, and the robustness of the collateral circulation (2, 3).

Consequently, not all LVO stroke patients are good candidates for endovascular thrombectomy, and, therefore, not all are treated by the same method (1). Thus, if the question of “how to treat LVO stroke patients?” seems to be answered as ET, although the technique may vary, the question of “which situations?” remains a problem, especially in those situations that are outside or underrepresented in the population of large trials, such as in cases of a low National Institutes of Health Stroke Scale (NIHSS) score.

In this terminal situation, the controversy persists. First, all low NIHSS scores are not equivalent with regard to clinical outcomes, which increases the variance of literature-reported outcomes (4). Second, present data have heterogeneous “low NIHSS score” definitions, LVO inclusion criteria, and primary endpoints, resulting in conflicting results in meta-analyses of the heterogeneous literature (5–8).

In this regard, we describe a patient with LVO stroke and low NIHSS presenting changes in collateral circulation routes by transcranial Doppler ultrasound (TCD) suggestive of the need for urgent treatment. Therefore, our objective was to suggest that monitoring collateral circulation routes can guide the selection of patients who will need ET.

## 2. Case description

**Figure 1:**
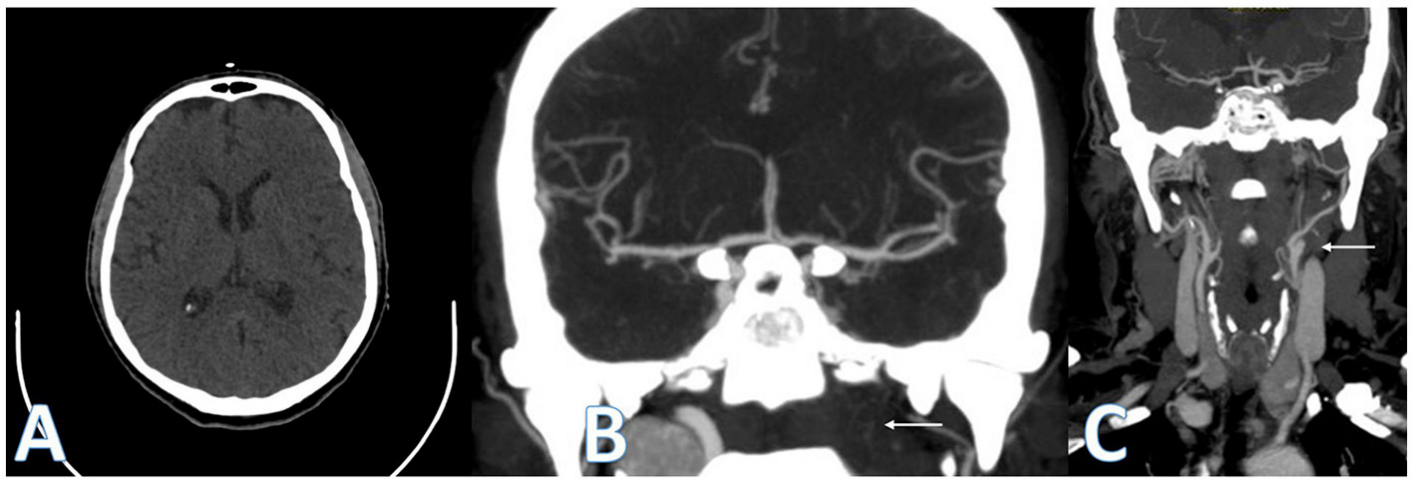
Computed tomography (CT). ASPECTS plan basal ganglia with no early ischemic changes **(A)**. Posteroanterior projection of CT angiography showing left ICA occlusion (white arrows) and flow to left MCA through ACOM **(B, C)**.

**Figure 2:**
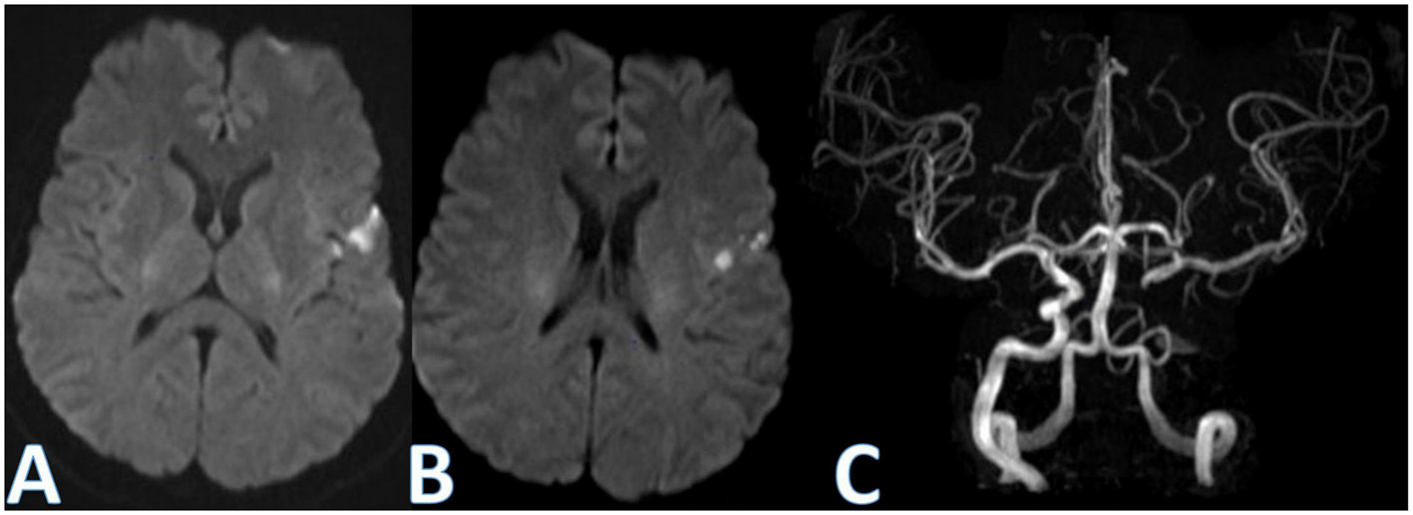
Diffusion-weighted magnetic resonance imaging (DW-MRI) and magnetic resonance angiography 3D time-of-flight (MRA 3D-TOF). DW-MRI showing left cortical diffusion restriction in MCA territory **(A, B)**. Posteroanterior projection of MRA 3D-TOF demonstrating left ICA occlusion with apparent patency of the circle of Willis by ACOM **(C)**.

**Figure 3:**
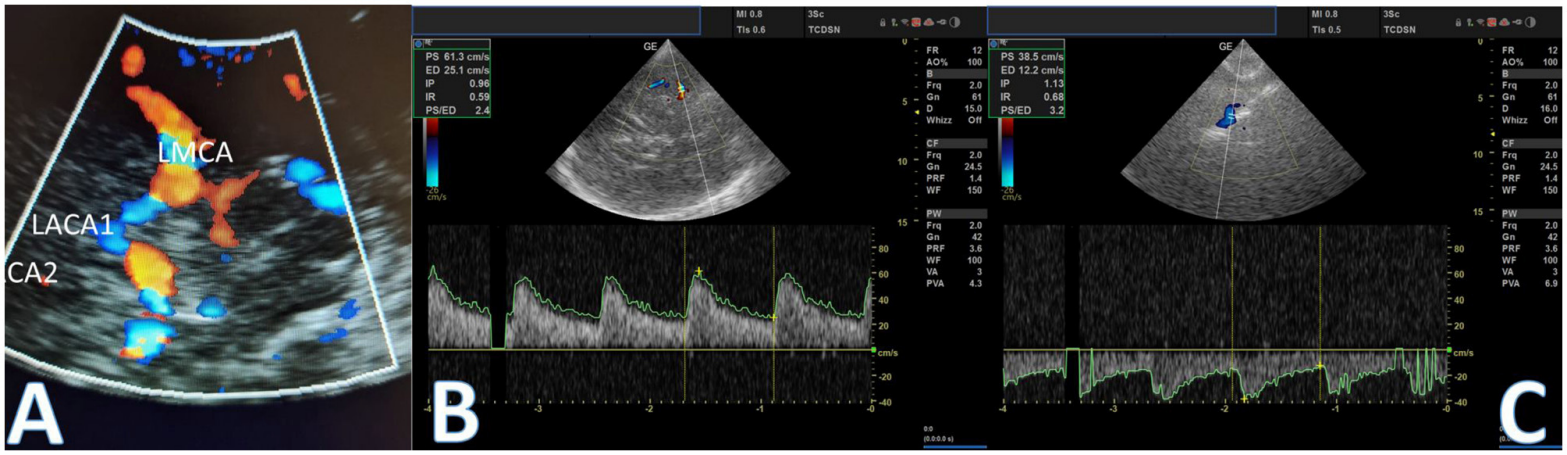
Transcranial color-coded duplex sonography (TCCS). TCCS shows the circle of Willis **(A)**. Initial evaluation shows reversed flow direction: left A1 segment flow from the ACOM (positive wave) **(B)** and second assessment after clinical worsening demonstrating flow inversion and a significant decrease in velocities **(C)**.

A 62-year-old man was admitted to emergency service presenting transient (<1 h) recurrent episodes of speech alteration, right hemiparesis, and numbness, with 6-h prior onset. His medical history included systemic arterial hypertension, diabetes, dyslipidemia, and smoking. On admission, the neurologic exam was normal (NIHSS 0). Computerized tomography (CT) and transcranial color-coded duplex sonography (TCCS) were immediately performed. Head CT showed microangiopathy without signs of intracranial bleeding (Figure 1A). Furthermore, CT angiography (CTA) of cervical and intracranial vessels evidenced left internal carotid artery (ICA) occlusion at the level of carotid bulb, with compensatory collateral flow from the circle of Willis by anterior communicating artery (ACOM) (Figures 1B, C). Furthermore, TCCS was performed and confirmed such findings (Figures 3A, B). Diffusion-weighted magnetic resonance imaging (DW-MRI) showed left cortical diffusion restriction in the middle cerebral artery (MCA) territory, and 3D time-of-flight magnetic resonance angiography (3D-TOF-MRA) confirmed left ICA occlusion with apparent patency of the circle of Willis by ACOM (Figure 2).

**Figure 4:**
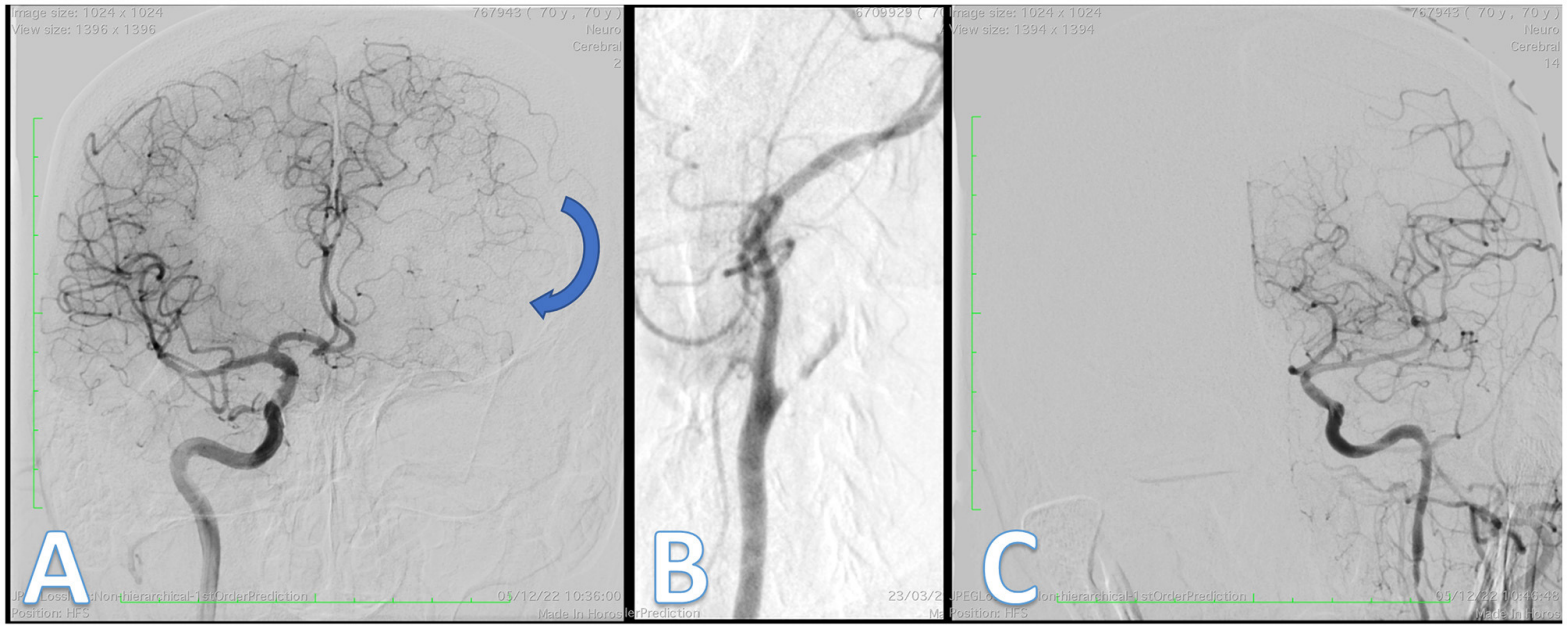
Digital subtraction angiography (DSA). Posteroanterior projection from right ICA shows pial collateral circulation (blue arrow) from left ACA to left MCA **(A)**. Posteroanterior projection shows left ICA occlusion with subocclusive stenosis **(B)** and final control from left ICA demonstrating complete reperfusion. There was no embolus in the anterior circulation **(C)**.

The patient underwent a stress test—walking a few minutes in the ICU—without deficits recurrence. Hospitalization in the intensive care unit was indicated, and aspirin plus clopidogrel was initiated. However, after ~8-h duration, the patient presented a new episode of right hemiparesis and speech alteration. A second TCCS showed a decrease >30% in flow velocity in the A1 segment of the left anterior cerebral artery (Figure 3C). The patient was then carried on with a diagnostic digital subtraction angiography (DSA), which revealed that cerebral left anterior territory was now supplied exclusively by anterior and posterior collateral pial circulation (CPC) (Supplementary Figures 1, 2), with failure in collateral flow from the circle of Willis (Figure 4A). Moreover, DSA revealed a left carotid bulb subocclusive stenosis, and revascularization was performed (Figures 4B, C). During the procedure, it was observed that the true lumen originated from the petrous segment leading to revascularization performed by two telescopic braided stents plus a carotid stent (Supplementary Figures 1, 2). The patient then fully recovered from deficits after the procedure and modified Rankin Scale score (mRS) 0 at follow-up after 3 months.

## 3. Discussion

This study reports on a patient who experienced an ischemic stroke with LVO and low NIHSS. Subsequent neurological deterioration and changes in collateral circulation pathways were monitored with TCD.

The cerebral collateral circulation can be classified into primary and secondary collaterals, with primary collaterals including arterial segments of the circle of Willis and secondary collaterals including the ophthalmic artery and leptomeningeal vessels (9). The primary collateral circulation network provides immediate blood flow to the affected area, while the secondary collateral network performs at a later stage. Anatomical variations in collateral vessels, capacity for vasodilation in response to ischemia, and individual risk factors influence the collateral recruitment process (9–13). The collateral circulation status in acute ischemia is a critical determinant of cerebral perfusion pressure and has important therapeutic and prognostic implications (14). Studies have shown that a negative pattern of collateral circulation is associated with worse functional outcomes, faster evolution, greater final volume of the ischemic core, and greater severity of disability upon admission (15–19). On the contrary, favorable collateral circulation is associated with higher rates of favorable functional outcomes in the third month after a stroke, reduced risk of intracranial bleeding during the endovascular procedure, and reduced 3-month mortality (20).

The assessment of cerebral collateral circulation can be performed using various methods, including computed tomography angiography, digital subtraction angiography, computed tomography perfusion, magnetic resonance imaging, and transcranial Doppler. A comparison between the utilization of these methods on acute stroke, particularly in LVO, is shown in Table 1. Therefore, TCD—a widely available non-invasive technique—can also assess cerebral collateral circulation (9). Recent studies have shown that the presence of secondary collaterals detected through TCD is a sign of insufficiency of the primary collateral system via the circle of Willis (9, 25). In a study of 70 patients with symptomatic carotid artery occlusion, those patients with collateral circulation through the ophthalmic artery or through the leptomeningeal vessels in addition to the circle of Willis had a worse cerebral hemodynamic state than the patients with collateral circulation only through the circle of Willis (25).

**Table 1:** Differences between CT-A, MRI, and ultrasound in acute stroke patients, particularly in cases of large vessel occlusion (LVO) (21–24).

**Imaging modality**	**CT-A**	**MRI**	**Ultrasound**
Type of image	Cross-sectional	Cross-sectional	Real-time, dynamic
Time to perform	Rapid (s)	Longer (20–60 min)	Rapid (min)
Sensitivity for detecting LVO	Moderate to high	High	Moderate
Sensitivity for detecting infarct	High	High	Low
Sensitivity for detecting hemorrhage	High	High	High
Contrast agent required	Yes	Sometimes	No
Availability	Widely available	Limited availability	Widely available
Cost	Moderate	High	Low
Safety considerations	Radiation exposure, risk of contrast-induced nephropathy	No ionizing radiation, risk of contrast-induced nephropathy	No ionizing radiation, no contrast agent

This case report describes a patient who presented a symptomatic carotid artery occlusion with a transient neurological deficit lasting <1 h. The occlusion was attributed to arterial disease in the left carotid bulb, diagnosed by CTA (Figure 1). The evaluation of collateral circulation was initially performed through CTA and TCCS, which revealed compensation of blood flow exclusively through the primary route via the circle of Willis. The circle of Willis is known to be the main collateral cerebral network, which can maintain flow in the middle cerebral artery (MCA) following internal carotid artery (ICA) occlusion within seconds (26). This system comprises the anterior communicating artery, which is considered the most potent collateral, and the posterior communicating arteries, which may supply blood flow in either direction between the anterior and posterior circulations (27). Indirect signs of collateral flow through the ACOM in patients with carotid artery disease may include decreased mean flow velocity and pulsatility in the ipsilateral MCA with the normal flow in the contralateral MCA, flow inversion in the ipsilateral A1 segment of the ACA, and increased blood flow in the A1 segment of the contralateral ACA, as well as in the ACOM (28). In this case, it was initially observed that the flow via the left A1 segment was inverted, indicating collateral filling via the anterior communicating artery. The patient underwent a stress test, which did not reveal a recurrence of symptoms, and was subsequently prescribed dual antiplatelet therapy. Endovascular treatment for diagnosed LVO was not performed.

After the initial presentation, the patient developed neurological deterioration, and simultaneously, a deterioration of the collateral circulation pattern was evidenced by TCCS. Unlike the initial assessment, TCCS demonstrated compensation of blood flow solely through the primary collateral route via the circle of Willis. However, following the neurologic deterioration, TCCS revealed indirect signs of secondary collateral recruitment via leptomeningeal circulation, as evidenced by a significant decrease in flow velocity >30% in the A1 segment of the left anterior cerebral artery. Additionally, as depicted in Figure 4A, the diagnostic DSA confirmed that the cerebral left anterior territory was now exclusively supplied by anterior and posterior collateral pial circulation. Patients with symptomatic carotid artery occlusion were assessed through TCD analysis of vascular reactivity utilizing carbogen inhalation. It was observed that those who exhibited collateral circulation through the ophthalmic artery or leptomeningeal vessels, in addition to the circle of Willis, presented a more compromised cerebral hemodynamic state than those with collateral circulation solely through the circle of Willis. Thus, the presence of these secondary collaterals indicates an inadequacy of the primary collateral system via the circle of Willis (25).

Following the loss of primary collateral circulation documented by TCCS and DSA, two initial hypotheses can be proposed. The first hypothesis is the presence of a second embolism in the A1 segment of the anterior cerebral artery territory. However, this hypothesis was not confirmed, as shown in Figure 4C, which showed no embolus in that territory. The second and more plausible hypothesis is that the patient has limited hemodynamic reserve due to contralateral carotid stenosis. Hemodynamic impairment in antegrade flow with corresponding collateral recruitment has been demonstrated in patients with luminal stenosis above 50%. Additionally, there is evidence that flow across the stenosis and into the downstream territory consistently decreases with regard to the degree of stenosis at various lesion sites (29).

In a study using advanced neuroimaging to assess collateral circulation failure, Campbell et al. suggested that non-invasive methods capable of repeatedly assessing collateral circulation could help understand its determinants and better select patients eligible for reperfusion treatment (18). Thus, with regard to therapy, the clinical worsening, and the hemodynamic changes in cerebral circulation evaluation, added to the left carotid bulb subocclusive stenosis revealed by DSA, motivated the endovascular revascularization. Considering the evolution of therapeutic methods to approach acute cerebral ischemia in the population of LVO patients, it is noticed that the degree of development of the patient's collateral circulation is of significant importance. The clinical outcome of patients who are undergoing intravenous thrombolysis and mechanical thrombectomy highly depends on the individual's collateral circulation status (14). According to McCarthy et al., this concept seems even more critical in the LVO and low-NIHSS population; they have suggested that these patients with a poor collateral profile are more susceptible to the early neurological deterioration (5). Our patient had carotid occlusion, and during the procedure, it was observed that the true lumen originated from the petrous segment. Therefore, revascularization was performed by two telescopic braided stents along with carotid stents (Supplementary Figures 1, 2). The presence of contralateral carotid stenosis, the patient's age, and risk factors for cerebrovascular disease led us to hypothesize an occlusion due to an atherothrombotic mechanism of the extracranial ICA.

An interesting issue that could be raised is the presence of tandem occlusion. However, during the procedure, no thrombus recovery was observed. Additionally, the initially performed non-invasive imaging method, CT angiography, did not reveal intracranial occlusion of branches of the anterior or middle cerebral arteries. Moreover, flow in the transcranial Doppler with an inverted pattern was observed in the anterior cerebral artery (Figure 3B). Finally, after the carotid revascularization procedure, the entire anterior circulation was open upstream, as shown in Figure 4C.

The importance of collateral flow in predicting stroke outcomes and response to treatment is increasingly recognized. Patients with poor collaterals may be more susceptible to early neurological deterioration and may benefit from early intervention, even with a low NIHSS score. In this report, the patient had compensatory collateral flow from the circle of Willis but presented a neurological worsening and failure in collateral flow, suggesting a need for urgent treatment. This highlights the importance of close monitoring of collateral flow and response to treatment in patients with LVO stroke. An intensive transcranial Doppler monitoring strategy could be useful in identifying patients who may benefit from endovascular thrombectomy. Transcranial Doppler can provide real-time information on changes in blood flow velocity and collateral flow patterns, allowing for early detection of neurological deterioration and prompt intervention. Further studies are needed to determine the optimal criteria for patient selection and the use of transcranial Doppler monitoring in guiding treatment decisions.

## Data availability statement

The original contributions presented in the study are included in the article/Supplementary material, further inquiries can be directed to the corresponding author.

## Ethics statement

Written informed consent was obtained from the individual(s) for the publication of any potentially identifiable images or data included in this article.

## Author contributions

ESN and ÍS: manuscript concept and writing. AV: manuscript writing and image design. IR, MB-A, and OP-N: manuscript revision. All authors contributed to the article and approved the submitted version.

## Supplementary material

Supplementary Figure 1 Digital subtraction angiography (DSA). Posteroanterior projection showing left ICA occlusion with partial recanalization in the intracavernous segment through external carotid artery anastomosis **(A)**. Oblique work projection showing microcatheter plus microwire in true vascular lumen **(B)**. Oblique intracranial injection showing intracranial vascular patency **(C)**. Final control after left ICA revascularization **(D)**.

Supplementary Figure 2 Digital subtraction angiography (DSA). Posteroanterior projection from left vertebral artery demonstrating posterior pial collateral circulation from left PCA to left MCA.
