# Supplementary figures and images for: Case report: Flow changes in routes of collateral circulation in patients with LVO and low NIHSS: a point favor to treat
Source: Front Neurol. 2023 Jun 8;14:1165484. doi: 10.3389/fneur.2023.1165484 (PMC10287161; doi:10.3389/fneur.2023.1165484)

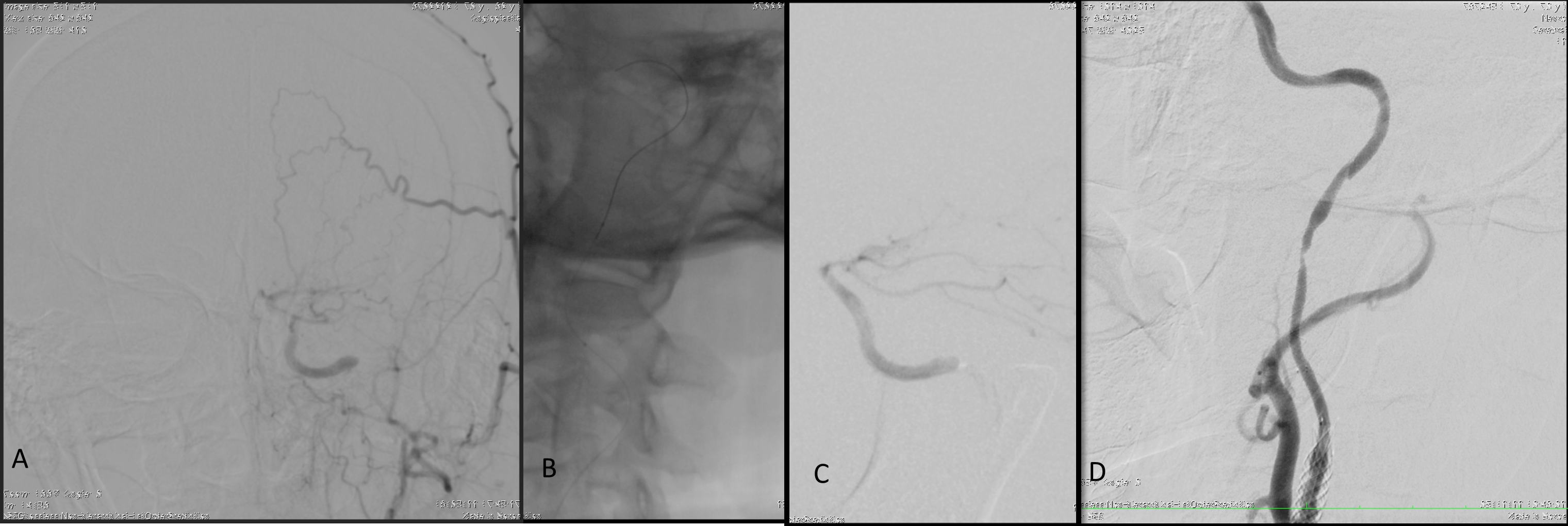

Supplement: Supplementary Figure 1 — Digital subtraction angiography (DSA). Posteroanterior projection showing left ICA occlusion with partial recanalization in the intracavernous segment through external carotid artery anastomosis (A). Oblique work projection showing microcatheter plus microwire in true vascular lumen (B). Oblique intracranial injection showing intracranial vascular patency (C). Final control after left ICA revascularization (D). [file Image_1.TIF]

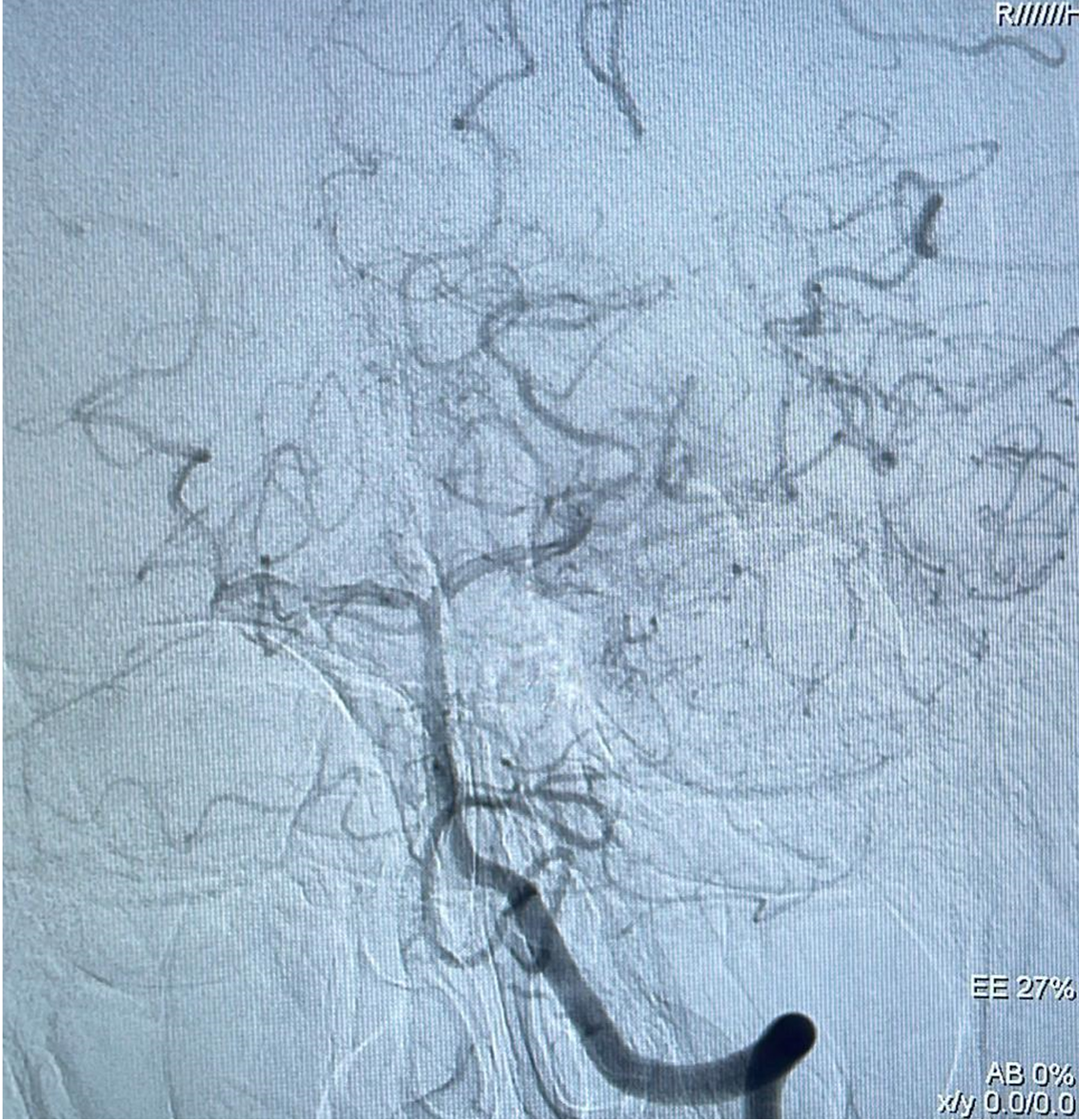

Supplement: Supplementary Figure 2 — Digital subtraction angiography (DSA). Posteroanterior projection from left vertebral artery demonstrating posterior pial collateral circulation from left PCA to left MCA. [file Image_2.TIF]
